# Epidemiological and clinical correlates of malaria-helminth co-infections in southern Ethiopia

**DOI:** 10.1186/1475-2875-12-227

**Published:** 2013-07-03

**Authors:** Andargachew Mulu, Mengistu Legesse, Berhanu Erko, Yeshambel Belyhun, Demise Nugussie, Techalew Shimelis, Afework Kassu, Daniel Elias, Beyene Moges

**Affiliations:** 1 School of Biomedical and Laboratory Sciences, College of Medicine and Health Sciences, University of Gondar, P.O. Box 196, Gondar, Ethiopia; 2 Institute of Virology, University of Leipzig, Leipzig, Germany; 3 Akililu Lemma Institute of Pathobiology, Addis Ababa University, Addis Ababa, Ethiopia; 4 School of Medical Laboratory Technology, Hawassa University, Hawassa, Ethiopia; 5 ACE Biosciences, Unsbjergvej 2A, Odense SOE 5220, Denmark

**Keywords:** Malaria, Helminthic infections, Co-infection, Ethiopia

## Abstract

**Background:**

In many areas of the world, including Ethiopia, malaria and helminths are co-endemic, therefore, co-infections are common. However, little is known how concurrent infections affect the epidemiology and/or pathogenesis of each other. Therefore, this study was conducted to assess the effects of intestinal helminth infections on the epidemiology and clinical patterns of malaria in southern Ethiopia where both infections are prevalent.

**Methods:**

A cross-sectional study was conducted in 2006 at Wondo Genet Health Center and Bussa Clinic, southern Ethiopia. Consecutive blood film positive malaria patients (N=230) and malaria negative asymptomatic individuals (N=233) were recruited. Malaria parasite detection and quantification was diagnosed using Giemsa-stained thick and thin blood films, respectively. Helminths were detected using direct microscopy and formol-ether concentration techniques. Coarse quantification of helminths ova was made using Kato Katz method.

**Results:**

The over all magnitude of intestinal parasitic infection was high irrespective of malaria infection (67% among malaria positive patients versus 53.1% among malaria non-infected asymptomatic individuals). *Trichuris trichiura* infection was associated with increased malaria prevalence while increased worm burden of helminths as expressed by egg intensity was associated with increased malaria parasitaemia which could be a potential factor for development of severe malarial infection with the course of the disease. Majority (77%) of the subjects had multiple helminths infection. *T. trichiura, Ascaris lumbricoides, Schistosoma mansoni,* and hookworm infestation accounted for 64.5, 57.7 %, 28.4%, and 12.2% of the infections, respectively.

**Conclusions:**

Populations in malaria-endemic areas of southern Ethiopia are multi-parasitized with up to four helminths. Mass deworming may be a simple practical approach in endemic areas in reducing the risk of severe malarial attack particularly for those at high risk of both infections.

## Background

Half the world’s population live in malaria-endemic areas, with an estimated 500 million clinical cases and over one million deaths annually [1]. Similarly, malaria is ranked as the leading communicable disease in Ethiopia, accounting for about 30% of the overall disability adjusted life years lost. Approximately 68% of the total population lives in areas at risk of malaria [2]. In Ethiopia, *Plasmodium falciparum* and *Plasmodium vivax* are the major parasites accounting for about 70% and 30% of infections, respectively, during peak transmission periods [3,4].

Soil-transmitted helminths (STH), on the other hand, are a group of common parasites that infect more than a billion people worldwide [5]. In Ethiopia, like other developing countries, infections with the major STH, including *Ascaris lumbricoides, Trichuris trichiura,* hookworm and *Schistosoma mansoni* are widely spread with variable prevalences [6-12]. In many areas of the world, malaria and STH are co-endemic, therefore co-infections are common, particularly in Africa [13,14]. The rate of malaria and STH co-infection in Ethiopia as reported in one study was 9.6%, 6.3% and 2.1% with hookworms, *A. lumbricoides* and *T. trichiura*, respectively [15]. In another study a 2.8% prevalence of urinary schistosomiasis and *P. falciparum* malaria was reported [16].

However, how concurrent infections affect the epidemiology and/or the pathogenesis of each other remains controversial [17-19] raging from increased severity of malaria to reduced severity and incidence of malaria during helminths co-infection [20-33]. The underlying reason for such different outcomes might be due to numerous factors, including differences in study design and case definition, data analysis and interpretation, the demonstration of antigen cross-reactivity between co-infecting organisms and host factors [20]. Studies on the impact of helminths on incidence, prevalence and severity of malaria in Ethiopia where both infections are hyperendemic is lacking. Therefore, this study was conducted to assess the effects of STH on the epidemiological and clinical patterns of malaria in a hyper-endemic area for both malaria and helminths infections.

## Methods

### Patients

This cross-sectional study was conducted at Wondo Genet Health Center and Busssa Clinic, Sidama Administrative Zone, South Ethiopia in a peak transmission season from November to December 2006. These areas are malaria endemic with high prevalence of helminthiasis. All patients presenting with signs and symptoms of malaria during the study period were included (N=230) and asymptomatic individuals with negative result for malaria (N=233). A pretested, structured questionnaire was used to collect socio-demographic and relevant clinical data of the participants.

### Diagnosis of malaria

Both thick and thin blood films were made in a single slide and were stained with Giemsa staining solution for detection and quantification of malaria parasites. To detect malaria infections, 200 fields (the equivalent of 0.5μl of thick blood film) were examined. The parasite density was expressed per μl of blood assuming 8,000 leucocytes per μl of blood. In brief, a thick fill was selected where the white blood cells were evenly distributed. Using the oil immersion objective, 200 white blood cells were counted systematically, by counting at the same time the number of parasites (asexual form only) in each field covered. Then the number of parasite per μl of blood was calculated by multiplying the number of parasite (asexual stages) counted against 200 leucocytes and 8,000 leucocytes and dividing the product by 200 [34]. After clinical examination and laboratory findings, patients were categorized in to different clinical forms of malaria (uncomplicated, severe and complicated cerebral malaria) following the national protocol [MOH, Standard Malaria Diagnosis and Treatment Guideline, 2004].

### Stool examinations

Malaria patients and their counter part controls were requested to deliver stool specimen. Those who volunteered to deliver the specimen were given a clean, dry, leak-proof plastic. Stool samples were then examined by direct stool examination method using normal saline. The portion of stool was processed by using the Kato Katz technique; about 3 g stool was preserved by 10% formalin for formol-ether sedimentation techniques. Coarse quantification of eggs was obtained by counting the number of eggs on a smear of 41.7 mg faeces, and a quantitative variable scoring (light infection/low worm burden, moderate infection/medium worm burden and heavy infection/massive worm burden) was created for each helminths following the standard procedure used by World Health Organization (WHO) [34,35]. Packed red cell volume was determined using the conventional methods following the standard protocol.

### Statistical analyses

The data were analysed using to SPSS version 13 statistical packages software. Intestinal parasite densities were transformed to log for analysis and geometric mean was used. Baseline characteristics and malaria parasites densities of groups with and without intestinal parasitic infections was compared by Student’s *t* test and ANOVA. Two-tailed *P* values was determined and considered significant when found <0.05. Logistic regression analysis was also used to determined association between variables.

### Ethical consideration

This study was conducted after institutional (Aklilu Lemma Institute of Pathobiology, Addis Ababa University) and regional (Southern Nation and Nationalist Regional State Health Department) ethical clearance was obtained and after written and/or verbal informed consent was obtained from the study participants. Participants who were found to be positive for any of the parasites were linked to the health institutions for treatment.

## Results

### Study subjects

A total of 230 patients with malaria infection and 233 malaria negative individuals were included. The mean (±SD, range) age of the subjects was not similar in both groups [17.8 (±14.15) versus 19 + 12.23. The majority (34.5%) of the participants were under nine years of age, and 54% of the subjects were males.

### Prevalence and intensity of helminths infection

The overall magnitude of intestinal parasitoses in both groups was 76.9% (352/463). The most frequent intestinal helminths diagnosed were *T. trichiura, A. lumbricoides, S. mansoni,* and hookworm with prevalence of 64.5% (227/352), 57.7% (203/352), 28.4% (100/352) and 12.2% (43/352), respectively. Amongst of those with helminthiasis, 77% (271/352) were having multiple helminths infection. Infection with two, three or four different species of helminths was observed in 37.5% (132/352), 22% (77/352) and 3.4% (12/352) of the participants, respectively. Out of the 271 participants with multiple infections, the frequent combinations of helminths were *A. lumbricoides-T. trichiura* in 28.8% (78/271); *A. lumbricoides-T. trichiura-S. mansoni* in 18.5% (50/271), and *A. lumbricoides-T. trichiura-*hookworm in 8% (22/271) of the patients. In 4% of the patients (11/271) four different helminths: *A. lumbricoides, T. trichiura, S. mansoni* and hookworm were observed. The prevalence of helminthic infection tends to reach its peak in the age group 10 to 19 years old followed by children of less than nine years old (Table 1). As indicated in Table 2, out of the 227 participants who were infected with *T. trichiura,* parasitic load was available for 224 patients. Accordingly, 172 (76.8%), 46 (20.5%) and six (2.7%) developed light, moderate and heavy infection, respectively. Similarly, out of the 203 participants infected with *A. lumbricoides,* 92 (45.3%), 96 (47.3%) and 15 (7.4%) developed light, moderate and heavy infection, respectively. Out of the 100 participants with *S. mansoni*, 51 (51%), 24 (24%) and 25 (25%) developed light, moderate and heavy infection, respectively. The intensity of intestinal worms expressed as geometric mean among the study participants for *A. lumbricoides, T. trichiura* and *S. mansoni* was 4,691, 486 and 112, respectively.

**Table 1:** **Prevalence and intensity of *Trichuris trichiura, Ascaris lumbricoides, Schistosoma mansoni *and hookworm in different age groups**

**Age (years)**	**No positive for parasites (%)**			**EPG † (corresponding 95 % CI)**			
	**Hw**	**Tt**	**Al**	**Sm**	**Tt**	**Al**	**Sm**
< 9	7 (16)	69 (30.4)	56 (27.6)	20 (20)	456 (351,651)	4,604 (27,577,686)	120 (65,223)
10-19	17 (16.3)	72 (31.7)	62 (30.5)	40 (40)	405 (289,567)	5,477 (32,479,234)	197 (121,320)
20-29	7 (16.3)	50 (22.0)	44 (21.8)	21 (21)	409 (266,627)	3,841 (20,777,102)	118 (63,219)
30-39	3 (7.0)	16 (7.0)	20 (9.8)	12 (12)	734(3,571,511)	5,078 (235,010,969)	108 (58,198)
40-49	4 (9.0)	10 (0.04)	12 (5.9)	3 (3)	560(1,152,709)	4,432 (125,415,667)	82 (8,825)
50+	5 (11.6)	10 (0.04)	9 (4.4)	4 (4)	356 (169,537)	4,717 (359,921,622)	50 (4,521)
	43 (100)	227(100)	203 (100)	100(100)	*P*† *=0.741*	*P*† *=0.971*	*P*† *=0.305*

*No*, Number, *Hw*, Hookworm, *Tt, T. trichiura, Al*, *A. lumbricoides*, *Sm*, *S. mansoni.*

*EPG†*, Eggs/g/faeces computed as geometric mean, *CI*, Confidence interval.

**Table 2:** **Intensity of infection for *T. trichiura, A. lumbricoides *and *S. mansoni *by age group and sex**

**Age**	**Status of Helminths infection §**											
	***T. trichiura***		***A. lumbricoides***							***S. mansoni***		
	**N (%)**		**N (%)**							**N (%)**		
	**Neg**	**light**	**moderate**	**heavy**	**Neg**	**Light**	**Moderate**	**Heavy**	**Neg**	**Light**	**Moderate**	**Heavy**
< 9	71	52	14	2	85	26	25	5	119	10	7	3
10-19	39	57	14	1	51	23	35	4	71	17	8	15
20-29	48	38	10	2	53	23	18	3	77	13	3	5
30-39	22	9	6	0	17	10	9	1	25	6	5	1
40-49	13	7	2	1	11	6	5	1	20	2	1	0
≥50	11	9	0	0	12	4	4	1	17	3	0	1
Total		172	46	6		92	87	15		51	24	25
Sex												
Male	108	98	25	3	122	49	55	8	173	32	14	16
Female	96	74	21	3	107	43	41	7	156	19	10	9

*Neg*, No intestinal parasites; **§** Infection status was defined according to classes of intensity used by WHO (WHO, 2002.

### Malaria infections

The distribution of malaria parasite by age and sex is summarized in Table 3. The majority 58.3% (134/230) of the malaria infections were due to *P. falciparum. Plasmodium vivax* accounted for 35.2% (81/230) of the cases. The rest, 6.5% (15/230) were mixed infections of *P. falciparum* and *P. vivax* (Table 3). Malaria parasite density was available for 186 of the patients. Accordingly, depending on the level of parasitaemia (expressed as number of parasites per μl blood), 84.4% (157/186) of malaria patients had a parasite density of less than 25,000 parasites/μl blood, 10.2% (19/186) had a parasite density between 25,000-50,000 parasites/μl blood and 5.4% (10/186) had a parasite density of greater than 50,000 parasites/μl blood (Table 4). However, only three had shown sign and symptoms suggestive of complicated malaria.

**Table 3:** Distribution of malaria parasite by age and sex

**Variables**	**Malaria infection**	***P. falciparum***	***P. vivax***	***P. falciparum *and *P. vivax***
	**(N=230)**	**(N =134)**	**(N=81)**	**(N=15)**
**Age**				
<9	88 (38.3)	40 (29.9)	41 (50.6)	7 (46.7)
10-19	59 (25.7)	39 (29.1)	17 (21.0)	3 (20.0)
20-29	50 (21.7)	33 (24.6)	14 (17.3)	3 (20.0)
30-39	19 (8.3)	13 (9.7)	4 (4.9)	2 (13.3)
40-49	6 (2.6)	3 (2.2)	3 (3.7)	0
≥ 50	8 (3.5)	6 (4.5)	2 (2.5)	0
**Sex**				
Male	130 (56.5)	75 (56.0)	46 (56.8)	9 (60.0)
Female	100 (43.5)	59 (42.0)	35 (43.2)	6 (40.0)
Total	230 (100)	134 (100)	81 (100)	15 (100)

**Table 4:** ***Plasmodium falciparum *parasitaemia (per μl of blood) in relation helminths infection status**

**Variable**	**Mean parasitaemia‡**	**95% CI**¶	**ß**¶	***P*†**
**Sex**				
Male	14,499	(6921,22078))	1.569	0.212
Female	24,832	(8305,41359)		
***T. trichiura***				
Light	19,446	(7604,31289)		
Moderate	31,500	(-265,63265)		
Heavy	39,763	(-8896,8842)	0.764	0.469
***A. lumbricoides***				
Light	12,892	(2828,22957)		
Moderate	14,001	(5313,22689)		
Heavy	29,023	(1085,56961)	0.639	0.531
***S. mansoni***				
Light	22,766	(1188,44343)		
Moderate	23,244	(-6561,53050)		
Heavy	37,232	(-19820,94284)	0.142	0.868

**‡** Arithmetic mean of *P. falciparum***; ß**¶= Coefficient of regression; *#CI*, Confidence interval;

†=P value.

### Malaria-helminth co-infection

The rate of helminths co-infection among the malaria patients was 67% (154/230). The magnitude of *T. trichiura, A. lumbricoides, S. mansoni* and hookworm among the malaria patients were 51.7% (119/230), 42.6% (98/230), 22.6% (52/230), and 10% (23/230), respectively. It was only *T. trichiura* infection that was significantly associated with malaria co-infection (P=0.002) (Table 5). As eggs per gram of *A. lumbricoides* increased, the chance of developing severe malaria attack increased showing a dose dependent risk of severe malarial attack (P= 0.035). Similarly, patients with heavy trichuriasis infection seemed to be at higher risk of severe malaria infections compared with light trichuriasis. However, this did not reach a statistically significant level (P=0.066). Both moderate and heavy infections of *S. mansoni* were also associated with severe clinical malaria infection though the difference was not statistically significant (P=0.067). Worm burden (expressed in helminths egg intensity) was associated with higher mean level of parasitaemia (number of parasite per μl blood) (P=0.032).

**Table 5:** Prevalence of intestinal helminths among malaria positive and negative individuals

**Type of helminth infection**	**Malaria infection**		**ß¶**	**OR (95% CI) #**	**P †**
	**Yes (N=230)**	**No (N=223)**			
*T. trichiura*					
Yes	119 (51.7)	108 (48.4)	0.308	1.361 (1.119,1.655)	0.002
No	111 (48.3)	115 (51.6)			
*S. mansoni*					
Yes	52 (22.6)	48 (21.5)	0.129	1.137 (0.976,1.326)	0.137
No	178 (77.4)	175 (78.5)			
*A. lumbricoides*					
Yes	98 (42.6)	105 (47.1)	0.137	1.147 (0.780,1.687)	0.147
No	132 (57.4)	118 (52.9)			
Hookworm					
Yes	23 (10)	20 (9)	0.083	1.086 (0.926,1.275)	0.086
No	207 (90)	203 (91)			
Any helminth					
Yes	154 (67)	198 (88.8)	0.402	0.669 (0.06,1.101)	0.669
No	76 (33)	25 (11.2)			

¶= Coefficient of regression; *OR*, Odds ratio; *#CI*, Confidence interval; †=P value.

## Discussion

Approximately 68% of the total population of Ethiopia lives in areas at risk of malaria [2]. According to the World Malaria Report in 2011, Ethiopia was categorized among the four countries with a 25 to 50% reduction in new malaria cases [4]. However, despite the continuous efforts made to control malaria during the past decade, infections with malaria remained high in many parts of the country [36]. The current study in Wendo Genet reflected the same notion, with a very high prevalence rate of malaria. Most of the malaria infections were caused by *P. falciparum* (58.3%) while *P. vivax* and mixed infections accounted for 35.2% and 6.5% of the infections, respectively. The persistently high prevalence of malaria urges a need for evaluation of the malaria control programme in the study area. In Ethiopia, *A. lumbricoides, T. trichiura,* hookworm, and *S. mansoni* infections are widespread with variable prevalence that may reach up to 90% in some places among the general population [6-12]. Likewise, the most predominant intestinal helminths diagnosed were *T. trichiura*, *A. lumbricoides, S. mansoni* and hookworm, with a prevalence of 64.5%, 57.7%, 28.4% and 12.2%, respectively. The high prevalence of helminthiasis in general and the predominance of specific parasites in the area were in agreement with previous reports in the region [8,37].

In many tropical areas of the world, malaria and STH co-exist with significant co-infection rates [13,14]. This was reflected by the high co-infection rates observed in the current study (67%) which is consistent with studies from other tropical countries from Africa and Asia [29,31]. Higher rate of severe malaria attack among individuals co-infected with helminths as compared with those free from helminths was the reported from Thailand [31]. However, African study from Uganda failed to show such association even in heavily infected individuals [29]. In this study, trichuriasis that was found to be significantly associated with increased malaria infection. *Schistosoma mansoni* was not associated with increased malaria infection in the current study. This, however, contradicts the report from Senegal where the incidence rate of malaria attacks was significantly higher among *S. mansoni*-infected individuals [17]. On the other hand, a study in Nigeria reported that periodic deworming of children with *A. lumbricoides* has protective effect from malaria infection [38]. This implies prior infection with *A. lumbricoides* is a risk factor for malaria infection. In line with this, although it did not reach to a statistical significance level, there was a high *A. lumbricoides-*malaria co-infection rate in the current study. However, it is difficult to strongly conclude the epidemiological interaction of helminths and malaria infection with such cross-sectional study. Hence, it would be useful to check the incidence of malaria in a longitudinal study among cohorts of people having/not having different helminthiasis with and without treatment. Conducting further studies on the co-epidemiology of malaria and helminths in Ethiopia could be of paramount importance to a potential public health value of synergistic opportunities for control.

The current study however, tried to determine the effect of helminths co-infection on the clinical manifestation of the malaria cases with only three cases presenting with sign and symptoms of severe malaria. The absence of severe malaria cases in highly ‘wormy’ malaria patients in the current study was, however, consistent with a study conducted by Nacher *et al. *[31] where helminths infection was associated with protection from cerebral malaria. However, a strong association between worm burden as expressed by egg intensity and malaria parasite density was observed which suggest that with the time course of the infection, such patients with high density of both helminths and malaria will tend to develop severe form of clinical malaria by influencing the immune response during co-infection [27,39]. Studies have reported a positive correlation between egg load of helminth infection and *Plasmodium* density [13,18]. In line with this, the current study found that individuals with light *T. trichiura* and *A. lumbricoides* infections had lower *Plasmodium* densities than those with moderate or heavy infection. Contrary to this, heavy *S. mansoni* infection was associated with lower *Plasmodium* densities [17]. However, the effect of specific helminths on the severity of malaria and the mechanisms leading to this needs to be determined in longitudinal studies in a larger pool of clinical cases of malaria.

## Conclusion

The majority of malaria patients in the current study were found to be multi-parasitized with up to four helminths. A strong association between worm burden as expressed by helminths egg intensity and malaria parasite density was observed which could be a dose-dependent risk factor for severe malaria attack with the course of the infection. Mass deworming may be simple practical approaches in endemic areas in reducing the risk of severe malarial attack particularly for those at high risk of both infections.

## Competing interests

The authors declare that they have no competing interests.

## Authors’ contributions

AM, ML, BE, TS, AK and DE conceived, designed and proposed the research idea. AM was involved in data collection. AM, ML, BE, DN, TS, AK and DE were involved in data entry, clearance, analysis and interpretation of the findings. AM, BM and YB were responsible for drafting the manuscript. All authors were involved in reviewing the manuscript and gave approval for publication.
